# Book Reviews

**Published:** 1822-06

**Authors:** 


### CRITICAL ANALYSIS

OF

### ENGLISH AND FOREIGN LITERATURE

RELATIVE TO THE VARIOUS BRANCHES OF

### Medical Science.

Quæ laudanda forent, et quæ culpanda, vicissim

Illa, prius, cretâ; mox hæc, carbone, *notamus*.—PERSIUS.

### FOREIGN.

HAVING by us several important foreign works, which we have announced for review, and which pressure of other matter have hitherto prevented us from duly noticing, we have determined to consecrate the whole of the reviewing department in the present Number to their consideration.

We feel the less reluctance in following this course, as no British work worthy of the attention of our readers has been published within the last few months on subjects connected with medical science.

ART. I.—*De la Physiologie de Systeme Nerveux, et specialement du Cerveau, &c.* Par M. GEORGET, D.M.P.—*On the Physiology of the Nervous System, and particularly of the Brain; with an Inquiry into the Nature of Nervous Diseases in general.* By M. GEORGET, M.D. 2 vol. 8vo. pp. 421, 432. Paris, 1821.

THE nervous system, whether in its healthy or morbid condition, has, of late years, become the subject of mature and scientific investigation. From GALL and SPURZHEIM down to the author whose work we announce, many writers of eminence, in England, France, and Italy, have, by their labours, thrown considerable light on a subject formerly very obscure, and almost wholly conjectural.

M. GEORGET is already advantageously known as the author of a treatise on Mania, which has been favourably received. The opportunities which this young physician has enjoyed, from his situation in the hospital of La Salpetriere, of studying that class of diseases, have enabled him to compose a work to which we are disposed to attach considerable importance. The present treatise is doubtlessly the result of similar observations, made under similar circumstances, on many very interesting subjects connected with the physiology of nerves, respecting which we are now, for the first time, put in possession of many authoritative explanations.

The present work is divided into two parts. In the first, by far the most extensive of the two, the functions of the brain and the nervous system are fully developed. In the second, we are favoured with some general ideas on the maladies of that system, and with particular descriptions of hysteria, hypochondria, epilepsia, and spasmodic asthma.

The classification adopted by Dr. Georget is by no means free from objections. Much of it is necessarily founded on gratuitous assumption; and although, in many parts of his work, the evidence of facts is brought forward in support of particular speculations, we are not sure that the manner in which he has treated the subject has not involved some of the most important individual questions into obscurity. At least, we have found considerable difficulty in seizing the right meaning of many of his assertions; probably from our inability of comprehending his aphoristic prolegomena.

Before entering on the physiology of the brain, the author gives some general considerations on the laws which govern nature,—examines, successively, the bodies presented to our observation,—and establishes the great distinctions which separate the two great classes of organic matter.

Thus, progressively, Dr. Georget arrives at that part of his treatise which embraces the consideration of the various functions of the nervous system. These he divides into TWO orders: the first consisting of those functions which are destined for the perception of impressions received through the nervous extremities, the formation of ideas, the manifestation of moral qualities, the transmission and execution of the determinations of our will; the second being those calculated to control the other organic forces of the animal system.

In his investigation of the intellectual functions of the nervous system, M. Georget endeavours to establish the proposition that the brain is the seat of the mental faculties, and that particular phenomena are produced by particular organs. M. Georget adduces the sentiments of several previous writers to prove that this doctrine is greatly anterior to GALL's, who ought to be considered as one of its promulgators, rather than its inventor. *Inventa perficere* nor *inglorium;* why, then, has not Professor Gall mentioned the author's names whose ideas he borrowed in forming his system of *cranioscopy?*

The cerebral functions are, according to our author, unequally developed in different individuals. The sex, age, and temperament, generally modify them; and all of them are dependent on the action of certain functional or sensorial excitants, which Dr. Georget has classed under the following heads; —1. *Climate and Season.* 2. *Profession.* 3. *Education.* 4. *Mode of Living.* 5. *Civilization.* 6. *Progress of Knowledge,* 7. *Religion and Superstition.* 8. *Political Institutions.*

There are various other modifications of the cerebral functions, which are observed during sleep,—dreaming, watchfulness, and somnambulism.

The second part of M. Georget's physiology of the nervous system, is consecrated to the study of its sympathetic relations. These relations, often neglected, but more often considered, incorrectly, as the causes of particular phenomena, are of the highest importance in pathology and therapeutics. They offer themselves to our view under two distinct orders:—1, those which connect the action of the nervous system with the various organs; and, 2, those which are referrible to the reaction of the organs on the nervous system.

The author commences with some general observations on sympathies. He denies the great influence attributed by the ancients to the abdominal organs over the encephalon.

It is curious to see a writer of M. Georget's acuteness attempt to establish, as sensations analogous to those of taste, sight, &c. four other modifications of the animal organization,—namely, thirst, hunger, the venereal appetite, and pain.

In the two sections which follow the preceding disquisition, the author considers the brain in its state of inactivity; and the application of the cerebral action to the purposes of therapeutics, constituting what has been called "moral medicine."

In this age, in which the most outrageous attempts have been made to establish a long train of sympathies as the agents of the majority of morbid phenomena observed during the short life of man, it will not be improper to consult the numerous remarks made by the author before us on a subject so much abused, and least understood by those who talk most loudly about it. M. Georget attributes a power of direct and reflected sympathy to each organ,—examines the plausibility of such a doctrine,—and, very wisely, limits the actuality of the thing within much narrower bounds than some of his contemporaries have done. Still he has *admitted* too much. The whole of it, in fact, is mere jargon,—a tissue of the most rank nonsense,—an easy method to cover ignorance! Do we understand a single fact dependent on nervous action? No. Every explanation hitherto attempted has been perfectly gratuitous,—unsupported, even, by the smallest particle of *presumptive* evidence.

We turn to the practical part of M. Georget's work. The pathology of nervous diseases in general first engages his attention. He acknowledges that we know very little of their nature; and that the resources of the healing art against them are neither numerous nor very effectual. He places the seat of these diseases in the brain and in the nervous system distinctly. He considers them as the result of irritation, excitation, and inflammation, of the nerves; in the same manner as these morbid actions have been observed to influence the other tissues of the body. Nervous diseases, referrible to any particular organ or viscus, according to the generally received opinion, are mere sympathies. The author endeavours to prove that hypochondriasis, hysteria, &c. are essential affections of the brain, and not of the digestive system, as some physicians have lately imagined. He contends that the most marked symptoms in these complaints are those which are purely referrible to the brain; and that the indications of a deranged stomach, &c. hold a very secondary rank during their progress. His descriptions of hypochondriasis, hysteria, and convulsive asthma, are so arranged that, while they adhere most strictly to truth, they, at the same time, afford a most prominent place to the cerebral symptoms of the disease.

Our readers will recollect that Monsieur Rostan had advanced an opinion tending to show that spasmodic asthma was dependent on an aneurism of the heart. This opinion M. Georget combats most stoutly; being himself inclined to believe that the disease may, and does, exist independently of any organic lesion either of the heart or of the lungs, and that it is principally produced by an identical morbidity of the nerves.

We might dilate further on these interesting volumes; but we conceive that we have said enough to excite the laudable curiosity of our readers.

---

Art. II.—*De Aure et Auditu Animalium aquatilium.* Auctore Ernesto Henrico Weber. 4to. Plates. Leipsic.

The anatomy of the ear of fishes is an object worthy the attention of comparative anatomists. Dr. Weber seems to have made a particular study of it; and, in the course of the work before us, he has brought forward several new facts, as the results of his assiduous inquiries.

These facts have been translated from the Latin, and inserted in a contemporary Journal, which, not being likely to fall into the hands of medical readers in general, would prove a very limited means of conveying, to the scientific public, the valuable information contained in Dr. Weber's work, were we not to repeat them in this place.

Such an enumeration of facts is, in reality, the best epitome we can give of the volume itself; the merit and the novelty of its contents consist wholly in them.

1. The *petromyzontes (lampreys)*, both of rivers and the sea, are furnished with a cartilaginous vestibulum separate from the cavity of the cranium, but they are destitute of semicircular canals, both cartilaginous and membranaceous. They are likewise destitute of lapilli enclosed in the vestibulum or in a bursa, and have no external organs of hearing. Their membranaceous vestibulum is divided into different cells.

2. In several genera of osseous fishes, and especially of the order abdominales, the swimming bladder is joined in a particular way with the internal ear, and is useful to the membrana tympani.

3. This conjunction of the swimming bladder with the internal ear in the cyprinus carpio *(common carp)*, brama *(bream)*, tinca *(tench)*, carrassius *(crusian)*, rutilus *(roach)*, aphyas, leuciscus *(dace)*, alburnus *(bleak)*, and, doubtless, in all the cyprini; likewise in the silurus glanis and cobitis fossilis, and the barbatula, is accomplished by means of six ossicula auditoria (three of which are placed on the right side, and three on the left,) united with the three superior vertebræ by articulation. These may be compared to the stapes, incas, and malleus. The apex of the malleus always adheres to the upper part of the swimming bladder.

4. All the fishes just enumerated are furnished with two cavities *(atria,)* situated in the first vertebra near the foramen occipitale. Each cavity is shut by the stapes of that side in which it is placed; and the stapes may either be drawn from it, or applied to it, by the action of the swimming bladder. Hence this cavity may be compared to the fenestra ovalis in man. Each cavity *(atrium)* is furnished with a little bone peculiar to itself, which shuts it up.

5. In all the fishes above enumerated, each cavity *(atrium)* has access to the sinus imparis by means of two holes cut in the occipital bone. This sinus is situated in the middle part of the basilary portion of the occiput. Passing into the cranium like a fork, it is divided into two canals, of which the right passes to the right labyrinth, and the left into the left labyrinth, to which it adheres in that place in which the saccus and vestibulum membranaceum are united.

6. In all the fishes above enumerated, there are found certain ostia leading into the same cavity of the cranium, covered with skin and muscles, which must be considered as the fenestræ of the osseous vestibulum, since the cranium of osseous fishes serves the same purpose as the vestibulum osseum.

7. In all these fishes, the three superior vertebræ, receiving the ossicula auditoria, are increased, augmented in size, and remarkably altered.

8. All these fishes possess an interior lapillus of the sac, remarkable by a peculiar form, long, and spinous.

9. The ossicula auditoria of the cyprinus are enclosed by two membranous auditorial fossæ, one of which is situated on the right side, and the other on the left side of the three superior vertebræ. The fossæ auditoriæ communicate with the cavity of the cranium by the two lateral occipital bones, and contain an oily liquor of the same nature as that in the cranium.

10. The ossicula auditoria of the cobitis fossilis are included in a cavity of the transverse process of the second vertebra, answering the purpose of the cavity of the tympanum.

11. The osseous capsule, enclosing the swimming bladder of the cobitis fossilis is formed from the transverse processes of the third vertebra, expanded into an osseous bulla. This capsule has two great external apertures, surrounded outwardly by an elevated margin, covered by the external cutis. By two other anterior openings, the apex of the malleus of the right and left side enters into the osseous capsule, and is there fixed to the swimming bladder. This osseous capsule answers the same purposes as the annulus of the tympanum in the human infant. Consequently, sonorous tremors have access to the swimming bladder through the apertures covered with skin; from which they are transferred, by means of the malleus, incus, and stapes, to the membranous labyrinth.

12. This conjunction of the swimming bladder and internal ear is brought about in other fishes, not by ossicula auditoria, but so that canals from the swimming bladder ascend directly to the head, and are joined to the ear in different ways.

13. In the sparus, salpa, and sargus, the top of the swimming bladder ascends to the base of the cranium divided into two canals. A peculiar membrane unites the apices of these canals to the margins of two oval bones situated on the right and left sides of the base of the cranium.

14. In the clupea harengus *(herring)*, two very narrow canals of the swimming bladder enter into two bony canals on the right and left side of the base of the occipital bone. Each of these canals is again divided into two small bony canals, whose extremities swell out into hollow bony globules, the anterior and posterior. The canals of the swimming bladder fill up these bony canals and globules. The appendix of the membranaceous vestibulum enters into the right and left anterior bony globule near the bullous end of the swimming bladder; so that, reaching the extremity of the swimming bladder, it forms a septum, which separates the cavity of the appendix of the vestibulum filled with water from the cavity at the extremity of the swimming bladder filled with air. The circumference of this septem is surrounded by a ring nearly cartilaginous. Hence, in the herring, the sonorous tremors of the swimming bladder are transferred to the membranous vestibulum itself.

15. The anterior part of the right membranous vestibulum of the herring communicates with the anterior part of the left membranous vestibulum, by means of a transverse membranous canal passing below the cerebrum, in such a manner that mercury cannot be injected into either vestibulum without filling, at the same time, the membranous vestibulum and the semicircular canals of the opposite side.

16. The lower end of the swimming bladder of the herring and anchovy is produced into a canal, situated between the two ovaries, and behind the rectum which opens into the ostium genitale.

17. The swimming bladder of the cobitis fossilis is not simple, but consists of two parts, the upper greater, and the lower very small, situated without the bony capsule. A fibrous process passes from the skin to the swimming bladder, by an external opening of the osseous capsule.

18. The canal for air *(canalis pneumaticus)* of the swimming bladder of the cyprini entering into the œsophagus, cannot be opened and shut by means of a valve, but forms a muscular tumor, through which the air-canal penetrates in a spiral direction, diminished to the fourth part of its size.

19. The ear of the ray-fish is not furnished with a single external passage, as all anatomists have hitherto believed; but with two. Besides the fenestra of the vestibulum cartilagineum closed by a membrane, described by Scarpa and Cuvier, there exists a fenestra of the vestibulum membranaceum, situated beside it. The fenestra of the vestibulum membranaceum is to be compared to the fenestra ovalis in man, and the fenestra of the vestibulum cartilagineum to the fenestra rotunda in man. The fenestra of the vestibulum cartilagineum forms an opening into the cavity of the vestibulum cartilagineum; the fenestra of the vestibulum membranaceum, in like manner, forms an opening into the cavity of the vestibulum membranaceum.

20. Between the fenestræ of the vestibula membranacea cut out in the cartilaginous cranium (belonging to the right and left ear,) and the skin covering the occiput, two bags are interposed, filled with a white calcareous liquor, and touching each other. From each of these a large membranous canal, entering through the fenestra of the vestibulum membranaceum, descends into the vestibulum membranaceum, and fills its cavity. These bags, called by Weber the sinuses of the external ear, and compared by Monro to the conchæ of the human ear, answer the purpose of the cavity of the tympanum; and the liquor included in them serve the purpose of the bones of the ear.

21. One or more very small canals, detected by Monro, but not found by Camper, Scarpa, Comperetti, and Cuvier, pass from the auditory sinus of each side to the cutis, and there open by very small mouths. By these canals, any excess of the calcareous liquid contained in the sinus auditorius may be thrown out.

22. There is a small muscle belonging to each auditory sinus, by means of which the auditory sinus may be compressed, and its liquor either thrown out through the small apertures opening in the cutis, or impelled through the canal into the membranous vestibulum. In this way the vestibulum membranaceum may be either compressed or relaxed.

33. The vestibulum membranaceum of the raia sarpedo marmorata *(risso,)* does not contain white cretaceous lapilli; but a gelatinous mass, mixed with a black-coloured sandy matter.

24. The membranous semicircular canals of the raiæ are joined to each other, and to the vestibulum membranaceum, in quite a different manner from those of the squalus carcharias *(white shark)*. The semicircular canals of the squalus carcharias have a semicircular form, while that of the raia has a circular form. The semicircular canals of the squalus carcharias proceed, by one extremity, from the vestibulum membranaceum, and, by the other extremity, return into it: whereas, those of the raiæ are quite separated from it, and indeed have no communication with it, except by two very small ducts. One of these ducts passes from the vestibulum membranaceum to the posterior canal, which has the form of a circle, and does not cohere to the remaining canals; the other passes to the anterior canal.

25. The observation of Treviranus, that the auditory nerves are not always to be considered as branches of the trigeminus, is proved to be true.

26. The nervi auditorii accessorii have different origins in different fishes; proceeding from the cerebrum, the par vagum, and the trigeminus. Nor are these nerves always destined to the same parts of the labyrinth. In the raia torpedo, the squalus carcharias and the petromyzon *(lamprey)*, the nervi auditorii accessarii do not belong to the ear. In several of the cyprini, the author describes a very remarkable deviation in these nerves.

27. The branches of the nervi auditorii belonging to the vestibulum are *soft*, and, as it were, spread over its inferior surface. The branches sent to the ampullæ are *hard*, and penetrate into the cavity of the ampullæ, constituting a semilunar plexus jutting out into the cavity. The sonorous tremors of the fluid included in the semicircular canals, are readily transferred to these nerves. The nerves of the vestibulum receive the sonorous tremors of the solid bodies included in the vestibulum, or sac.

**Art. III.**—*Recherches sur l'Inflammation de l'Arachnoide Cerebrale et Spinale, ou Histoire theorique et pratique de l'Arachnitis.* Par MM. Parent, Duchatelet, et L. Martinet, M.D.—*Researches on Inflammation of the Spinal and Cerebral Arachnoid Membranes; or a theoretical and practical History of Arachnoiditis.* By MM. Parent, &c. &c. 1 vol. 8vo. pp. 605. Paris, 1821.

The plan of this work is both simple and easy of comprehension. After a short, but exact, description of the arachnoid membrane, the authors proceed to explain its functions in the healthy state, and next the phenomena of its morbid appearances.

In the second chapter, the history of arachnoiditis is minutely detailed; and the disorganization produced by that disease is described with perspicuity; its physiological pathology, or the theory of its symptoms, are fully exposed; and, lastly, the therapeutical means necessary to be adopted during its treatment.

The third section consists of clinical observations on cerebral arachnoiditis, collected by the authors at the bed-side of the patients, or from the authority of many physicians of well-known celebrity. The third chapter contains the history of spinal arachnoiditis, with some observations on that disease.

In all the cases mentioned as having terminated fatally, which are by far the most numerous, the necroscopic examinations are added; and the degree of disorganization produced is compared with the particular symptoms noticed during the progress of the disease; a circumstance which renders these cases highly interesting. Some of the examinations have taken place on patients, who, having been cured of arachnoiditis, died, subsequently, of a different malady. In these cases, a description of the organic alterations which have occurred, and their relation to the symptoms observed during life, are given, and afterwards contrasted with the symptoms noticed in those cases of arachnoiditis which terminated fatally.

Upon these observations the authors have constructed a number of tables, exhibiting, at one view, many interesting circumstances; such as the influence of external causes, the sex, and period of life attained by the patient on the accession of the disease; its proportional duration; the relation of these proportions to the age of the patient; the regions of the arachnoid most commonly affected; and the connexion of the latter with the period of life at which the complaint appeared; the frequency of certain symptoms,—such as those in which the organs of vision or of motion are concerned; and the relative connexion of these with the region affected by inflammation. Care has been taken to note every case followed by suppuration. Each table is framed upon a number of observations, amounting to from 116 to 118; all of which are so completely detailed, that no circumstance, either with regard to the disease or the examination, has been omitted, which might invalidate the conclusions that may be deduced from them.

With respect to the exciting causes of arachnoiditis, the authors observe, that percussion, and especially violent concussions, have produced the disease in several instances. In some cases, the disease appeared to follow melancholia, metastasis, or suppressed exanthemata. Two cases only are mentioned as having been produced by insolation; while, in one instance, arachnoiditis was found complicated with hydrophobia. The proportions, in reference to the male and female sex, are two to one. With regard to the age of the patients, the authors have divided the epochs of life into the following orders:

1st. Infancy, subdivided into three periods;—viz. from birth to the age of five years: from five to eight: from eight to fourteen years.

2d. Adolescence, into two periods;—from fifteen years to twenty-one: and from twenty-one to thirty.

3d. Adults, into two;—from thirty-one to forty: and from forty to sixty.

4th. Old age;—from sixty-one to eighty years.

Adopting these divisions, it appears that, upon 116 cases, forty-nine occurred in adolescents, thirty-eight in adults, and twenty-nine in infants. Only five cases were observed among old people. In the infantile cases, there were five patients under five years of age; fifteen, between nine and fourteen years; the rest between five and nine. In the two divisions of adolescence, thirty-four cases occurred between the fifteenth and thirty-first year. The number of adults is equally divided.

The duration of arachnoiditis is uncertain: in most cases, however, it terminates on the seventh, ninth, or the eleventh day. On some rare occasions, it has been prolonged to the thirty-first. If the different durations of the disease be compared with the ages of the patients, it will appear that the more acute attacks increase, in a progressive proportion, with the ages between five and sixty years; but, as the duration does not always exhibit the same phenomena, the authors have considered it necessary to divide it into three periods; the first of which they call that of *excitement*, characterized by cephalalgia, which is generally circumscribed, deep-seated, and fixed, sometimes accompanied with nausea, and a febrile state not always very apparent: while in the second, or the inflammatory, the pain becomes stronger, and is usually accompanied with a spasmodic action of the muscles, particularly of the thoracic members; and often with disorder of the intellectual faculties.

The third period is designated by the appellation of *collapsus;* the character of which is a general debility, a comatose state, abolition of the senses, and loss of motion by paralysis, either local or general.

When the attack of arachnoiditis is very severe, it is often impossible to distinguish these periods. The disease then proves almost inevitably fatal. When, on the contrary, it comes on gradually, the first symptoms are so little apparent, that they are often unnoticed for a considerable time. It is on this account that MM. Parent and Martinet have minutely exposed them, insisting principally on those symptoms which accompany the invasion of the disease; the only period of the attack which affords a chance of success. These symptoms are then continued down to the termination by death; which is followed by the investigation, *post mortem*, of the alterations sustained by the organs in consequence of arachnoiditis. These morbid alterations consist in increased redness, thickening and opacity of the membranes; in exudations covering its surface, which are gelatinous, sero-gelatinous, purulent, and sero-purulent; likewise in false membranes, effusions, and adhesions.

It is to be regretted that, with all the accuracy of the authors before us, satisfactory conclusions cannot always be drawn, in reading their work, on the importance of the symptoms in facilitating the diagnosis of this awful malady. Does this uncertainty arise from the nature of the facts themselves; or is it that the examples brought forward are not sufficiently numerous? At all events, it is apparent that no established rule can be advanced with certainty; a proposition which can easily be proved by making a rapid analysis of that part of the work which relates to the physiological pathology of the complaint. Dilatation of the pupils does not bear constant and essential relation to effusion, either in the ventricles or in any other part of the brain and spine. The same may be said with regard to their contraction. The rolling of the eyes may be considered as indicative of suppuration of the arachnoid membrane. Strabismus, in some cases, has coincided with it. Trismus and distortions of the mouth often accompany arachnoiditis, without giving any positive indication of this disease. The presence of coma cannot be attributed exclusively to effusion. Cephalalgia occurs in all disordered actions of the arachnoid; its particular seat does not point out that of the disease. Delirium is common with young and robust subjects, in arachnoiditis of the convex hemispheres, previous to suppuration; but is never known in arachnoiditis of the base. Hemiplegia generally happens when effusion has taken place on the opposite side; but it is not always produced by it. Contraction, convulsions, and rigidity, form no characteristic symptoms; and vomiting, the common result of the sympathy existing between the stomach and brain, is not specially connected with any modification of the disease.

That part of the work which speaks of the treatment is, without exception, the best, on account of the extensive and minute investigations which the authors have entered into. The utility of cold affusions is clearly demonstrated, and the mode of application directed in a concise manner; the accidents that may happen subsequently to their use, are described; and the proper method to obviate them is mentioned. A strict injunction is laid down respecting the period of convalescence from this most critical disease, during which the patients should be particularly recommended to be abstinent.

In detailing their clinical observations, the authors attempt to establish those principles by which practitioners ought to be guided in their mode of treatment. External causes are least of all to be neglected; more especially insolation and percussion. We ought always to be on our guard on the appearance of cephalalgia, however slight it may appear. Arachnoiditis, produced from external causes, is generally followed by suppuration, which, spreading over the whole membrane affected, renders the operation of trepanning perfectly useless.

It is of no moment, in the treatment of arachnoiditis, to ascertain the exact portion of that membrane which is affected; but it is necessary to acquire a correct diagnosis of the disease, that the chances of its favourable or unfavourable termination may be the more accurately calculated.

Inflammation of the convex portion of the membrane mostly happens in adults; that of the base is most common among children. Delirium forms the essential character of the former, and is, in the third period of the disease, replaced by coma; while the latter is marked especially by coma, unattended by any other disorganized functions of the intellectual faculties. With infants, it presents a complication of spasmodic with comatose symptoms. A tetanous reversion of the head backwards, accompanied with contraction of the vertebral column, and pain in that region, indicates the presence of spinal arachnoiditis. But no essential symptom has been observed, which may lead to distinguish arachnoiditis of the ventricle from that of the base.

It is to be observed, that serous effusion on the convex surface does not,—while, on the contrary, a purulent effusion does —produce paralysis in most cases: yet no direct connexion can be established between effusion, paralysis, and convulsions. Arachnoiditis is generally found combined with some other disease: it seldom exists alone, and is very rarely connected with apoplexy. This remark is by no means a new one.

Several observations are advanced to prove that arachnoiditis assumes an intermittent form; and cases of necroscopic examinations are adduced to support the correctness of its diagnosis. Finally—and this should be the great object of all medical treatises—the authors prove, by facts, that, when the disease is attacked in time and by rational means, the prospect of success is very encouraging. Amongst the means recommended by the authors, bleeding and the cold affusion are considered as the most important and successful that can be had recourse to.

It seems to have been the object of the authors, in the work before us, to collect a great number of cases, carefully avoiding those of a doubtful nature or that are incomplete; and, by comparing them together, to draw from them the most reasonable conclusions. In this they have been successful. We shall terminate our short analysis by giving the following extract from the work itself, expressive of the principles upon which the treatise has been conducted.*

"Dans l'examen et le resumé de ces observations dont nous pouvons garantir l'exactitude, ayant eu soin d'écarter toutes celles qui nous ont paru tant soit peu defecteuses, nous nous sommes scrupuleusement attaché a bannir tout systême et toute hypothèse, afin de ne donner que le resultats des faits: ainsi dans les objets que nous traitons, nous exposons avec franchise ce qu'il y a d'inconnu, ce qu'il y a de faux, ce qu'il y a de douteux, ce qu'il y a de certain. Si cette marche est monotone et peu attrayante, elle a, du moins, l'avantage d'être conforme à celle qui est adoptée dans toutes les sciences physiques; et puisqu'il est reconnu que ces sciences lui doivent les progrès immenses qu'elles ont fait depuis quelques années, pourquoi ne l'adopterait on pas en médecine, cette belle partie de l'histoire naturelle, qui dois marcher de pair, maintenant, avec les sciences exactes?"

---

Art. IV.—*Association Intellectuelle. Méthode Progressive et d'Association, ou de l'Art d'Etudier et d'Operer dans toutes les Sciences, et particulièrement en Médecine; suivi d'une Clinique générale Interprétative des Phénomènes Morbides, et spécialement des Maladies des Couches.* Par L. V. F. Amard, d.m. ex-Chef du Service Medico-Chirurgical de l'Hôpital General de la Charité de Lyon, &c. 2 vol. 8vo. Paris, 1821.

It not unfrequently happens to reviewers, to look into the concluding part of a voluminous publication, for the only materially useful portion of its contents. Imposing as the title

---

* We have, for a very obvious reason, adopted the term *arachnoiditis*, instead of *arachnitis*, proposed by the authors. The former, in fact, means what the disease really is,—a state of inflammation of a something like a *tela araneæ*; the latter implies inflammation of the *aranea* itself, which is absurd.—A. B. G.

of the work before us may sound, its importance will be found concentrated in the few words by which that title is terminated. M. Amard is a man of no inconsiderable talent; but, in giving way to that love of declamation, new-fangled synonymes, and obscure phraseology, which so clearly marks the writer of defective education and bad taste of the present day, he has exposed himself to obloquy and ridicule.

We said that the only important part of M. Amard's work is that which is announced by the concluding sentence. We have, in fact, been pleased at the collective information he has adduced on puerperal maladies; and we consider his observations on this point as likely to rescue the work itself from total oblivion.

M. Amard pretends that there are, yet, three great *desiderata* in medecine:—1st, a correct method of studying that *science*; 2d, a general interpretative clinic of morbid phenomena; and, 3d, a special clinic for puerperal diseases. These *desiderata* have existed for the last twenty centuries; and Mr. Amard now comes forward, at last, to supply them. "Je viens, après deux mille ans, combler ces trois lacunes."

Let us see how he has proceeded in doing it.

The first part of the work is entitled "De l'Art d'Etudier la Médecine actuelle;" and, as every thing with our author must be metaphorical, the precepts he wishes to inculcate on this subject are addressed to a friend, under the garb of "Conseils à Ariste." The principal advice is, "to observe first and read afterwards." M. Amard is not very friendly disposed towards authors in general, and does not readily admit the utility of books,—in which he seems more wise than cunning; since, what would become of his two ponderous volumes, if such a doctrine against books obtained generally? But it is not only the want of good writers that M. Amard laments; he also deplores the absolute deficiency of critics in the performance of their office.

"Il est trop vrai que les critiques ne sont que des méchancetés où le cœur prend plus de part que l'esprit et la raison. Quand donc paraîtra-t-il un Aristarque, qui se dira: j'ai fait des ouvrages, et je me sens capable de juger ceux des autres; je les jugerai selon mes lumières et ma conscience; j'assignerai à chaque auteur la place que je lui croirai sincèrement méritée; je lirai les livres du même genre, s'il en est, qui ont été publiés, et je marquerai le rang que celui du nouvel auteur doit occuper parmi eux; je fermerai l'oreille aux instigations de la haine et aux sollicitations de l'amitié pour n'écouter que la voix d'une exacte justice; enfin, je procéderai avec tant d'attention et d'impartialité, que mes œuvres puissent être consultées par mes contemporains comme règle de ce qu'ils doivent croire ou penser sur les productions nouvelles, et que, loin de périr en naissant, comme périssent les critiques, qui s'évanouissent avec la passion qui les soutint ou les dicta, ces mêmes œuvres soient avouées de la postérité, et reconnues d'elle pour d'irrévocables jugemens. Cet Aristarque, Ariste, est encore à trouver."

In the second part, we have a description of the various systems of medicine, pourtrayed, as usual, in a figurative style. The author imagines a number of medical men, himself included under the name of Aristeus, assembled to discuss the superiority of their own individual system of medicine over that of every other. Dealeis is a courtier, who attends only patients in high life, and places the whole secret of the practice of medicine in an intuitive knowledge of complaints in the physician, and his luck in being successful or unsuccessful, greatly in vogue or for ever forgotten.—Deeply initiated in every system, both ancient and modern; having read every work of importance on the question; and versed in all the branches of human knowledge, Critias thought he could have no system of his own.—Lenorius, successively adopting every system, and laughing at every one of them; despising the labours of the ancients, and pitifully looking upon the exertions of his contemporaries, was considered as a man of genius. Some called him a systematic man; others, eccentric; a third class, a madman: and they were right.—Simo, after having long and unmercifully criticised every physician, had, at last, done the same with regard to medicine itself, and considered this science to be a mere tissue of futile speculations.—Euxenius, endowed with great penetration, endeavoured to show that medicine had no fixed principles, because it was studied, taught, and practised, without method. Lastly, Aristeus, (the author himself,) "homme d'un profond savoir, né pour tout ce qu'on peut imaginer de grand et de noble," delivers his own sentiments, and proceeds to detail his system; the development of which occupies the succeeding hundred and forty pages, besides eighty more, which are taken up in comparing the said system with every other system, constituting the *third part* of what the author has called the filling-up of the " première lacune."

The order in which M. Amard proceeds to supply the other two *desiderata*, is the inverse of that which he had announced in his preface; the *special clinical* medicine of puerperal maladies being the second, instead of the third, object of consideration in the present volumes.

M. Amard, having had the superintendence of the public Lying-in Hospital at Lyons, has availed himself of the many opportunities he thereby had in the course of his practice of noticing the various diseases to which women are liable, in the pregnant as well as in the puerperal state. From these notes, and the recorded details of cases, he has compiled the second great division of his work. He has arranged his observations under the heads of Natural Labours, *(couches naturelles;)* Varieties of Natural Labours, *(varietés des couches naturelles;)* and Complicated Labours, *(couches compliquées,)* or labours preceded, attended, or followed, by diseases incidental to that state. These diseases are treated under different heads:

1. Engorgemens ou commencement de depot.
2. Engorgemens froids.
3. Avortemens.
4. Hemorrhagies.
   *a.* During, and at different periods of, pregnancy.
   *b.* After labour.
5. Nervous symptoms caused by the labour.
6. Inflammation consequent on taking emmenagogues to promote abortion.
7. Inflammation in consequence of using violence in terminating the labour.
8. Putrefaction following the death of the child *in utero* during pregnancy.
9. Total prostration of strength at the time of labour.
10. Accidents in consequence of a retained placenta.
11. Inflammation, and various operations owing to a vitiated condition either of the pelvis or of the head of the fetus.

Of the forty or fifty cases which illustrate these various points of puerperal medicine,—all of them particularly interesting, and accompanied by sound reflections,—we shall merely extract the following; trusting that the example may serve as a warning to young practitioners in midwifery, who, with a little smattering of knowledge of the art, picked up in lecture-rooms, boldly adventure to use instruments, which, to our knowledge, have, in more than one case, been productive of irreparable mischief. We are often, at the two lying-in institutions to which we belong, made the unwilling depositaries of secrets reflecting on the unskilful practice, the rashness, and the no less blameable ignorance of certain practitioners in midwifery and midwives, that would make one shudder at the danger to which puerperal women are often exposed by their attendants. The author's reflections on the following case will be found not a little applicable to the state of midwifery in this country.

"*Inflammation gangréneuse des parties génitales, suite de violences faites par des accoucheurs ignorans.*

"Une fille de vingt-deux ans, bien constituée, ne pouvant accoucher naturellement, eut le malheur de tomber dans des mains ignorantes. Elle était aux douleurs depuis deux jours, lorsqu'on se détermina à extraire l'enfant en le retournant d'abord, en appliquant le forceps ensuite, et enfin, en le saisissant avec des crochets. Ces manœuvres furent continuées sans relâche, par trois officiers de santé, toute une nuit durant, et sans aucun succès. On apporta cette fille à l'hospice. L'enfant présentait la face dans la troisième position ; je le retournai sur le champ ; et l'accouchement fut terminé en moins de quelques minutes, car le bassin était bien conformé. L'enfant était mort ; la tête mutilée par le forceps, et perforée par les crochets. Le même jour, l'accouchée eut une soif ardente, et le ventre extrêmement douloureux. Le lendemain, elle rejetait, par le vomissement, tout espèce de boisson, et rendait involontairement les urines et les matières ; le ventre se tendit fortement. Le surlendemain, il était météorisé, moins douloureux ; elle avait des nausées continuelles, et vomit, à plusieurs reprises, une eau aigre et glaireuse. Elle perdait, par le vagin, un sang noirâtre et infect. Elle parlait beaucoup, frappée de la terreur d'une fin prochaine. Sur le soir, l'œil était égaré, et la face couverte, çà et là, de grosses gouttes de sueurs ; les prommettes étaient d'un rouge ardent, et le reste du visage d'une pâleur livide ; elle avait la respiration haute, rare, et courte ; le pouls intermittent, s'évanouissant sous le doigt ; elle mourut dans la nuit.

"Elle prenait une infusion de fleurs de mauves et des potions calmantes. On la fomentait avec des anodins.

"*Nécropsie.*—La matrice, le vagin, et jusques aux grandes lèvres étaient gangrénées ; l'épiploon et les intestins phlogosés, et leurs vaisseaux apparens et comme injectés de pourpre. La cavité du péritoine contenait une certaine quantité d'eau sanguinolente. L'estomac et les intestins étaient dilatés par des gaz. Quant à la symphyse du pubis, elle avait supporté de si énormes efforts, qu'elle s'ouvrit, d'elle-même, lorsqu'on incisa les tégumens qui la recouvrent.

*Remarques.*—Encore un exemple de mort causée par l'impéritie de gens que nos lois qualifient du nom d'officiers de santé ; et il y en aura bien d'autres. Si quelque chose doit surprendre, sous le régime médical où nous vivons, ce n'est pas de trouver l'art de guérir tombé en de telles mains; c'est au contraire de voir des médecins illustres, honorant leur pays de leur œuvres et de leur nom, et recevant de ce pays la flétrissure d'une patente, contineur leur travaux ! Et cependant ils font bien ; il est des grands hommes d'obliger les ingrats.

"Par quelle négligence toutefois et quel outrageant dédain, les réglemens concernant l'exercice de la médecine, sont-ils encore assez imparfaits, pour que de pauvres mères de famille soient exposées à tomber dans les mains homicides de pareils ignorans ! que dis-je ? des statuts en médecine, de l'ordre ! eh ! si quelques figures héraldiques valent plus qu'un grand homme ? n'importe ; le genre humain est là, éternellement, et c'est pour lui qu'il faut travailler : un jour viendra qu'il connaitra ses bienfaiteurs, et qu'il saura les honorer. Les barbares ! ils n'ont pas trouvé encore de place et de récompense nationales à décerner aux savans !"

The remaining portion of the first, and the whole of the second volume are filled with cases of modifications of the ordinary diseases, when occurring in the puerperal state, accompanied by many remarks of the author. This is the part of M. Amard's work which he has styled "Clinique generale interpretative des phenomenes morbides dont les femmes en couche sont atteintes; comme celles qui ne sont pas accouchées," and which is intended to fill the third *lacune* in the practice of medicine, complained of by the author. In this division of the work, several cases are brought forward illustrative of the author's peculiar views respecting various classes of disease affecting puerperal women. His observations are wholly confined to the female patients received, in a pregnant state, in the general hospital of La Charité of Lyons; and it appears that none but girls of the town are admitted. This being the case, our readers will not be displeased to learn, from the annexed table, the number of those unfortunate beings that were received yearly in that establishment, in an advanced state of pregnancy, for the space of six successive years. The information is highly curious; and the column marking the prevalence of fatal cases among them is calculated to enhance the value of this document.

| Date. | No. of Girls of the Town admitted. | Of whom died. |
|---|---|---|
| 1806 | 508 | 12 |
| 1807 | 483 | 7 |
| 1808 | 501 | 15 |
| 1809 | 550 | 7 |
| 1810 | 568 | 5 |
| 1811 | 551 | 8 |
| | 3191 | 54 |

In this large number of prostitutes, in a pregnant state, admitted in the general hospital of La Charité, there were three of fifteen years of age, six of sixteen, thirty-three of seventeen, eight of from forty-five to forty-eight, one of fifty-four years of age; some between thirty-five and forty-five; but the largest proportion of between twenty and thirty.

No one can question, we have reason to believe, the accuracy of the author before us in his statements of various diseases and their treatment; and, assuming this to be the fact, we cannot help expressing a wish that, like LAMOTTE or Dr. PERFECT, M. Amard had limited himself to a collection of cases illustrative of his particular points in practice. We should then have had a valuable stock of useful information; and not have to regret that sound judgment or accurate discrimination did not influence M. Amard in the choice of his subjects.

**Art. V.**—***Anatomie de l'Homme; ou, Description lithographiée de toutes les Parties du Corps Humain.*** **Par MM. Beclard et Jules Cloquet. Publiée par M. C. de Lasteyrie, Editeur. 1ere, 2de, 3me, 4me, 5me, 6me, and 7me livraison, grand in f°. Paris, 1821-22.**—***Human Anatomy; or, Lithographed Description of all the Parts of the Human Body.*** **By MM. Beclard and Jules Cloquet. Published by Lasteyrie. Paris, 1821-22. 7 Numbers, large folio.**

We have already announced the appearance of this original work, from the pen and the pencil of two of the best anatomists of the present age, in one of our preceding Numbers; and begged to direct the attention of our readers to its great merits and superiority over every other treatise of general Anatomy that has appeared in this or any other country during the last twenty years. Without entering, on the present occasion, into the details of the various subjects treated in the first seven numbers lying before us, which are not susceptible of being analyzed, we shall merely point out of what importance we consider Messrs. Beclard and Cloquet's Anatomy to be to medical practitioners; state the measures that have been adopted by the authors to ensure to it every possible degree of perfection; and mention the terms on which the work is to be sold to the public.

We possess, already, several anatomical works, illustrated with plates, executed with more or less skill and precision. Of these, some are distinct treatises on particular subjects of anatomy, and do not answer the purpose of general instruction in this branch of medical science; others, on the contrary, are more general in their nature, extending to every part of human anatomy; but, with some of them, the plates are imperfect or ill executed, while the really good are scarce, and to be had only at an extravagant price.

These reasons have induced M. Lasteyrie to undertake a publication, the execution of which he has placed in the hands of Messrs. Beclard and Cloquet, for the express purpose of supplying the student in medicine, as well as the senior practitioner, with a guide and reference to anatomical knowledge; which, to a degree of accuracy in the graphic delineation of the parts, on a large scale, should combine clear descriptions and the merit of cheapness. That he has succeeded in his views most completely, we have no hesitation in asserting; and we predict a high degree of popular favour to his undertaking.

MM. Beclard and Cloquet have successively treated the subjects of Osteology, Myology, the Organs of Senses, Neurology, Angiology, Splanchnology, and Embryology. In regard to the plates, the editor has secured the services of two eminent artists, who seem to have made anatomy their principal study.

We have seldom seen so much precision, even in the minutest details, combined with so much neatness and distinctness in the outlines. It not unfrequently happens, even to the most expert draughtsman, when engaged in tracing the representation of anatomical objects of a delicate nature, and requiring the strictest precision, to find some of the minor lines, on which the truth of the whole figure often depends, vanish, as it were, from under their pencil; unless directed in their performances by the watchful suggestions of a professor well versed in anatomy. To avoid this source of error, each delineation, in the work before us, is taken from nature, and under the immediate superintendance of one or both of the eminent anatomists already mentioned. To judge from the result of these united efforts, we should say that the lithographic art has never been more successfully, as well as usefully, employed; and that the work before us reflects great credit on the French nation.

The work is published in numbers, containing six plates each and three sheets of letter-press, and will be completed in forty numbers, at the very moderate price of nine francs, or seven shillings and sixpence, which is not the least of its recommendations.

---

Art. VI.—*Memoire sur l'Auscultation Appliquée a l'etude de la Grossesse; ou recherches sur deux nouveaux signes propres a faire connaitre plusieurs circonstances de l'etat de Gestation. Lu à l'Academie Royale de Medicin, dans sa seance generale du* 26 *Decembre*, 1821. Par M. J. A. Lejumeau de Kergaradeck, &c. Paris. 8vo. 1822.

Every attempt to improve the art of midwifery must, at all times, be welcomed by us, who are firmly convinced of its present defective state. The manner in which this most important branch of the medical profession is taught in this country has been justly condemned by a very sensible writer in one of the early numbers of the Quarterly Journal of Foreign Medicine, as nugatory, imperfect, and tending to promote mischievous presumption, rendered more fatal by inevitable ignorance, rather than a thorough knowledge of the true principles of the art. How, in fact, can any pupil be expected to meet the exigency of difficult cases of parturition when left to his own resources, if the only instruction he has received in midwifery is that which he has been able to pick up during his attendance to two or three courses of written lectures, or in seeing the simulated representation of labour on a leathern puppet?

Instruments are indeed applied on this automaton by way of tuition, and the pupil is told something about turning in cases of hemorrhage, convulsion, flooding, or an arm presentation. But how is either operation performed in the lecture room? The student pokes about the clumsy blades of a short forceps within the leather manikin in his endeavours to seize the ill-concealed head of a fœtus; or, having introduced his right-hand, agreeably to instructions, seizes, without any further direction, the first limb he can hook his finger upon, and drags it to view, when he perhaps finds, to his great horror, that the limb is the corresponding arm?* or if he has been fortunate enough to bring down one of the pelvic limbs, the next which he has drawn forth has been the other arm!† We were present at the examination of a poor woman who died a few hours after delivery with the forceps, when it was found that a horrid gap had been made through the vagina between the bladder and the womb! We have a preparation of the womb of another poor woman whom we were called to examine after death subsequently to delivery with instruments, and in whom we found the parts lacerated in sundry places, and in a state of absolute gangrene! But to what purpose do we multiply these awful examples of stubborn ignorance and presumptuous interference, unless it were with the hope of calling the attention of the legislature to the necessity of regulating the practice of midwifery, which, as things are now, *may* be in the hands of the most ignorant pretender? What can be more flagrant in the way of abuse of the public confidence in matters of obstetricism, than the case of a person beginning obstetrical practice without having attended a college, an hospital, or a lecture room; calling himself a physician accoucheur into the bargain, to assume more importance; and lecturing on the art he himself has learned in so ambiguous a manner? Can the fair sex think themselves secure and well attended by such a practitioner, if such an one exist? And yet the case *may* be so, for aught that the legislators of the land, or the guardians of the public health, care about the matter.

With regard to midwives, the thing is still worse. Some have applied to us to be admitted to practice in one or other of the two lying-in charities to which we belong, who could not positively spell or read their own name, nor pronounce one of the names of the parts connected with parturition; and who, on the strength of having attended *one* course of oral lectures, received a diploma or certificate of being perfectly qualified to undertake the office of midwife! Some of these said certificates

---

* A fact, *me teste superveniente*, both in the lecture room and in the lying-in chamber!

† A fact again, *me teste*. A gentleman, now a lecturer, gave us once a drawing of a case which had happened to a country practitioner, who had been a pupil in London, where both arms and the left foot had been brought down into the vagina. We witnessed an instance of no less ignorance in a young boy man-midwife, turning for the first time in his life, because he had been taught how to do it, where the child had been turned not on his belly, but on his back, and the spine dislocated!

were signed by persons affixing the initials of M.D. to their name, to which by law they are not intitled, for want of proper education! we have hitherto performed a painful duty in regard to all certificates thus signed, and have refused them as invalid, in imitation of the salutary resolution lately passed by the Apothecaries' Company—that no candidate will be admitted for re-examination, whose certificates are signed by pseudo-M.D.'s, viz. from the St. Andrew's or Aberdeen colleges.

These very important questions, on which, we regret to see, the profession have wilfully shut their eyes, are of a greater moment than the public are at first view inclined to imagine. The general results of midwifery practice in this country on some few occasions in which such results have been published, have served as a matter of triumphant contrast to our continental neighbours! Are not, then, the lives of the best part of the population, as well as the honour of the nation, interested in the question? Look at the tables published by Chaussier, and by him contrasted with those published by Dr. Bland and Dr. Merriman. What a lesson in the comparison!

We have so far digressed from the immediate consideration of the production before us; because we have long been impressed with the conviction that it is the bounden duty of every regular practitioner in midwifery to promote its advancement and respectability; and we felt desirous of expressing our sentiments on this occasion—sentiments which we know are shared alike with the very many highly respectable accoucheurs practising in this polished metropolis.

M. De Kergaradec is not a practitioner in midwifery; but he comes forward with a twofold discovery, to which we confess he attaches more importance than the thing itself seems to deserve, supposing for a moment, that the discovery itself proves not to be a delusion.

Accident led M. Kergaradec to ascertain that by employing the means so successfully adopted by M. Laennec, with regard to the exploration of pectoral diseases, not only the pulsations of the heart of the fœtus in utero may be distinctly heard at any time between the period of quickening and the end of gestation; but the spot on which the placenta is implanted may be detected from similar perceptions of the vibration of the umbilical vessels, and of the wheezing noise made by the blood circulating through that spongy body.

Several examples are adduced in which these points were decidedly ascertained by this method in the presence of, or by, indeed, some very eminent practitioners, among whom was Laennec himself, who vouchsafes for the accuracy of M. Kergaradec's assertions. And the reporter to the Royal Academy of Medicine, to whom the consideration of the memoir was referred, having repeated the author's experiments and observations, agree in drawing from them the same conclusions.

The operation is performed either by applying the ear to different parts of the abdomen, or by means of Laennec's instrument, which latter mode must be decidedly preferable on many accounts.

The practical inferences from this discovery are enumerated by the author in the following order, though with more development than we can afford space to give them.

1. It furnishes us with an additional character in the diagnosis of gestation; and is, therefore, so far likely to be useful in certain cases of female disease, difficult labour, or of judicial medicine.

2. From the number and strength of the pulsations, it will enable us to judge of the strength and health of the fœtus.

3. If, in the case of twins, the pulsations should, by future observations, be found to be double, the birth of twins will be easily predicted.

4. The precise knowledge of the spot on which the placenta is implanted may, in some instances, be found of considerable utility, particularly in the case of performing the Cesarean operation.

5. Auscultation will also be of service in indicating the existence of extra-uterine pregnancies. For when the pulsations belonging to a fœtus are heard in a part of the abdomen where they are not usually found, and an examination, *per vaginam*, has enabled us to ascertain the non-development of the womb, the conclusion of an extra-uterine fœtation will not be far removed from truth.

It should be observed, that M. Maior, a surgeon at Geneva, had, long before M. Kergaradec, mentioned the possibility of ascertaining whether a fœtus in utero, in a state of total rest, was alive or dead, by applying the ear to the abdomen, as, in the former case, the pulsation of its heart would be distinctly heard by the practitioner.

Having thus brought before our readers this new source of research, on which great noise is now made in Paris, we leave it to them either to laugh at it, or to receive it with proportionate respect.

